# Downregulation of Plasma miR-215 in Chronic Myeloid Leukemia Patients with Successful Discontinuation of Imatinib

**DOI:** 10.3390/ijms17040570

**Published:** 2016-04-15

**Authors:** Kazuma Ohyashiki, Tomohiro Umezu, Seiichiro Katagiri, Chiaki Kobayashi, Kenko Azuma, Tetsuzo Tauchi, Seiichi Okabe, Yutaka Fukuoka, Junko H. Ohyashiki

**Affiliations:** 1 Department of Hematology, Tokyo Medical University, Tokyo 160-0023, Japan; patchsei@yahoo.co.jp (S.K.); tauchi@tokyo-med.ac.jp (T.T.); okabe@tokyo-med.ac.jp (S.O.); 2 Department of Molecular Science, Tokyo Medical University, Tokyo 160-0023, Japan; t_umezu@tokyo-med.ac.jp (T.U.); chiaki-k@tokyo-med.ac.jp (C.K.); 3 Department of Molecular Oncology, Institute of Medical Science, Tokyo Medical University, Tokyo 160-0023, Japan; k_azuma@tokyo-med.ac.jp (K.A.); junko@hh.iij4u.or.jp (J.H.O.); 4 Department of Electrical Engineering, Kogakuin University, Tokyo 163-8677, Japan; fukuoka@cc.kogakuin.ac.jp

**Keywords:** plasma miR-215, chronic myeloid leukemia, imatinib, discontinuation

## Abstract

Approximately 40% of chronic myeloid leukemia (CML) patients who discontinue imatinib (IM) therapy maintain undetectable minimal residual disease (UMRD) for more than one year (stopping IM (STOP-IM)). To determine a possible biomarker for STOP-IM CML, we examined plasma miRNA expression in CML patients who were able to discontinue IM. We first screened candidate miRNAs in unselected STOP-IM patients, who had sustained UMRD after discontinuing IM for more than six months, in comparison with healthy volunteers, by using a TaqMan low-density array for plasma or exosomes. Exosomal miR-215 and plasma miR-215 were downregulated in the STOP-IM group compared to the control, indicating that the biological relevance of the plasma miR-215 level is equivalent to that of the exosomal level. Next, we performed real-time quantitative RT-PCR in 20 STOP-IM patients, 32 patients with UMRD on continued IM therapy (IM group) and 28 healthy volunteers. The plasma miRNA-215 level was significantly downregulated in the STOP-IM group (*p* < 0.0001); we determined the cut-off level and divided the IM group patients into two groups according to whether the plasma miR-215 was downregulated or not. The IM group patients with a low plasma miR-215 level had a significantly higher total IM intake, compared to the patients with elevated miR-215 levels (*p* = 0.0229). Functional annotation of miR-215 target genes estimated by the Database for Annotation, Visualization and Integrated Discovery (DAVID) bioinformatic tools involved cell cycle, mitosis, DNA repair and cell cycle checkpoint. Our study suggests a possible role of miR-215 in successful IM discontinuation.

## 1. Introduction

Tyrosine kinase inhibitors (TKIs), including imatinib (IM), are now a standard frontline therapy for chronic myeloid leukemia (CML) that yields successful outcomes [1]. CML therapy is widely accepted, and achieving cytogenetic and molecular responses at certain time points after TKI therapy and switching among TKIs have been proposed as practical guidelines [2]. In addition, clinical trials for TKI discontinuation are still on-going, with the most famous studies being the STIM study [3] and the TWISTER study [4]. In the STIM study, approximately 40% of CML patients with a molecular remission of at least a two years’ duration safely discontinued IM; they maintained undetermined minimal residual disease (UMRD) for more than two years, and 60% maintained major molecular response (MMR: MR^4.0^) [3]. The STIM study showed that male gender, low Sokal score and IM duration of >72 months were associated with maintaining treatment-free remission [3]. The TWISTER study, using a patient-specific primer for BCR-ABL, revealed that some CML patients who maintained UMRD after stopping IM had BCR-ABL DNA, leading to molecular relapse [4]. Early reduction of *BCR-ABL1* transcription by TKIs induces a deep molecular response [5], and expansion of cytotoxic natural killer (NK) cells [6] could serve as a predictive marker for future treatment-free remission.

We have reported that extracellular microRNAs (miRNAs), including plasma miRNAs and exosomal miRNAs, are altered in hematopoietic neoplasias [7,8,9], and some support malignant progression via the microenvironment [10,11]. miRNAs are noncoding single-stranded RNAs of 21–25 nucleotides that have recently been implicated in the regulation of cellular processes, such as apoptosis, proliferation, development or differentiation, not only in normal hematopoiesis, but also in hematological malignancies. The biological and clinical implications of cellular miRNAs are now being extensively studied. Recently, extracellular miRNAs, also known as secretary miRNAs, have been proposed as having multiple functions, including immune-modulation, angiogenesis and cancer progression [11,12,13]. Extracellular miRNAs, such as exosomal miRNAs, are extensively studied in non-hematologic diseases, such as cardiovascular diseases, endocrine disorders or pulmonary diseases [12]. Unlike solid tumors, the cellular component is easily obtained in the context of leukemia; however, analysis of the cell-free fraction, including plasma, is worthwhile in cases of complete remission when neoplastic cells are not present in the peripheral blood. We attempted to identify circulating miRNAs in CML patients who maintained UMRD after stopping IM (STOP-IM), and we studied target molecules by using bioinformatics tools.

## 2. Results

### 2.1. miRNA Expression Profiling by the TaqMan miRNA Array

**Figure 1:**
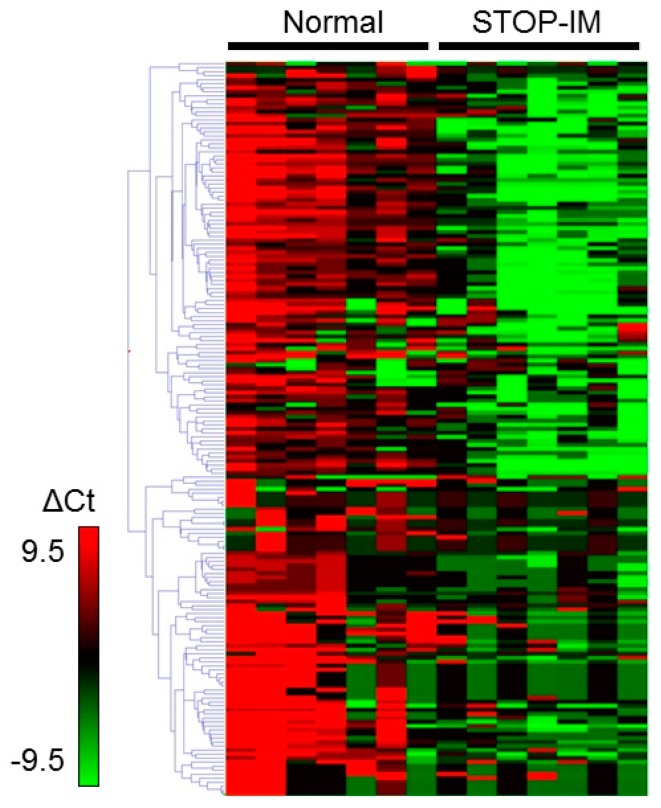
miRNA profiling by the TaqMan (Thermo Fischer Science, Waltham, MA, USA) miRNA array. A differential expression pattern was found between stopping imatinib (STOP-IM) patients and control subjects. Using Sequence Detection System (SDS, Version 2.4, Thermo Fisher Science) and DataAssist software (Thermo Fisher Science), we calculated the expression of miRNAs based on their *C*_t_ value normalized by the *C*_t_ value of ath-miR-159. Data were analyzed with GeneSpring software (Agilent Technologies, Santa Clara, CA, USA).

To identify candidate plasma miRNAs with altered expression in the STOP-IM group, we screened miRNA expression using a TaqMan miRNA array on seven unselected CML patients from the STOP-IM group and seven healthy volunteers. Between these two groups, we observed differential expression of 69 miRNAs identified by using GeneSpring software (Agilent Technologies, Santa Clara, CA, USA) (Figure 1; Gene Expression Omnibus (GEO) Accession No. GSE75392). Based on the Wilcoxon rank sum test of the R statistical software, only two miRNAs, miR-215 (*p* = 0.006841) and miR-134 (*p* = 0.028805), had greater than a 1.5-fold change in expression.

To determine whether miRNA expression in plasma indeed reflects exosomal miRNA, we compared the expression profiles by using a TaqMan low-density array. We randomly chose three patients in the STOP-IM group and three healthy volunteers. Eleven miRNAs in plasma and 35 exosomal miRNAs were found to be significantly different between the two groups. Among these miRNAs, downregulation of miRNA-215 expression was highly significant in the STOP-IM group (plasma miRNAs, *p* = 0.00311 (Table S1); exosomal miRNAs, *p* = 0.00039 (Table S2)); therefore, we concluded that plasma miR-215 expression mirrors exosomal miRNA expression and focused on expression of this miRNA for further study.

### 2.2. Quantification of Individual miRNA by Real-Time Quantitative Reverse Transcriptase-Polymerase Chain Reaction

**Figure 2:**
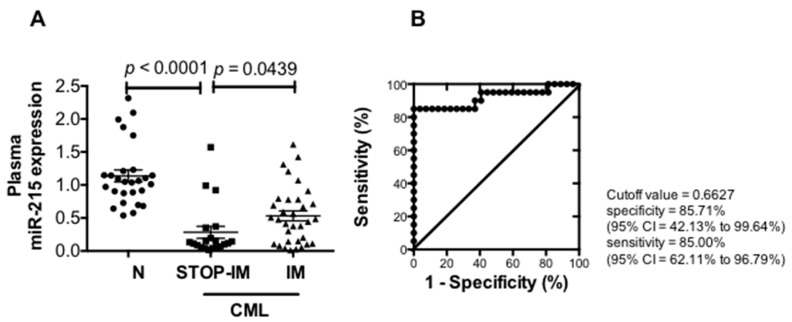
Expression of miR-215 (**A**); The cutoff level for miR-215 expression between the STOP-IM and control groups (**B**). The area under the curve level was 0.9204. The cutoff value was thus determined to be 0.6627, which was used in the following analyses of plasma miR-215 expression. Abbreviations: IM, imatinib mesylate; CI, confidence interval; CML, chronic myeloid leukemia; STOP-IM, patients with sustained undetectable minimal residual disease (UMRD) for more than six months after discontinuing IM; N, healthy volunteers; IM, CML patients taking IM who have a molecular response.

Since the expression of miR-215 was typically downregulated in both the plasma and exosomes of the STOP-IM group compared to healthy individuals, we analyzed the plasma miR-215 expression level in CML patients by individual real-time quantitative RT-PCR. Plasma samples were obtained from 20 patients in the STOP-IM group and 32 of the IM group, as well as from 28 healthy volunteers. A validation analysis using a large number of samples revealed that expression of miR-215 was significantly lower in the STOP-IM group than in the healthy volunteers (*p* < 0.0001) or in the IM group (*p* = 0.0439) (Figure 2A).

### 2.3. Clinical and Biological Relevance of miR-215 Expression

To estimate a cutoff value for miR-215 expression in plasma for distinguishing between STOP-IM and healthy control groups, we performed receiver operating characteristics (ROC) curve analysis (Figure 2B). The area under the ROC curve was 0.9204% with 85.00% sensitivity (95% confidence interval (CI), 62.22%–96.79%) and 85.71% specificity (95% CI, 42.13%–85.71%). The cutoff value was thus determined to be 0.6627, which was used in subsequent analyses of miR-215 expression. We next analyzed the clinical relevance of the plasma miR-215 expression level in CML patients who sustained MR^4.0^ while on IM (IM group). There were no significant differences in age (*p* = 0.5903), sex (*p* = 1), Sokal score (*p* = 0.2719) or prior interferon therapy history (*p* = 0.7026). Notably, the patients with a low plasma miR-215 level had a significantly higher total IM intake, compared to the patients with a high miR-215 level (*p* = 0.0229), while no significant differences existed with regard to the daily IM dose (*p* = 0.395) or duration of IM therapy (*p* = 0.0871) between the two CML groups with or without a low expression level of plasma miR-215 (Table 1). These two CML groups also did not significantly differ in the International Scale at the time of plasma miR-215 examination (*p* = 0.4126). The duration of UMRD from CML diagnosis (*p* = 0.3827) and the duration of stable molecular response (maintain MMR) (*p* = 0.7548) did not differ between these two groups (Table 1).

### 2.4. Functional Annotation of miRNA-215 Target Genes

**Figure 3:**
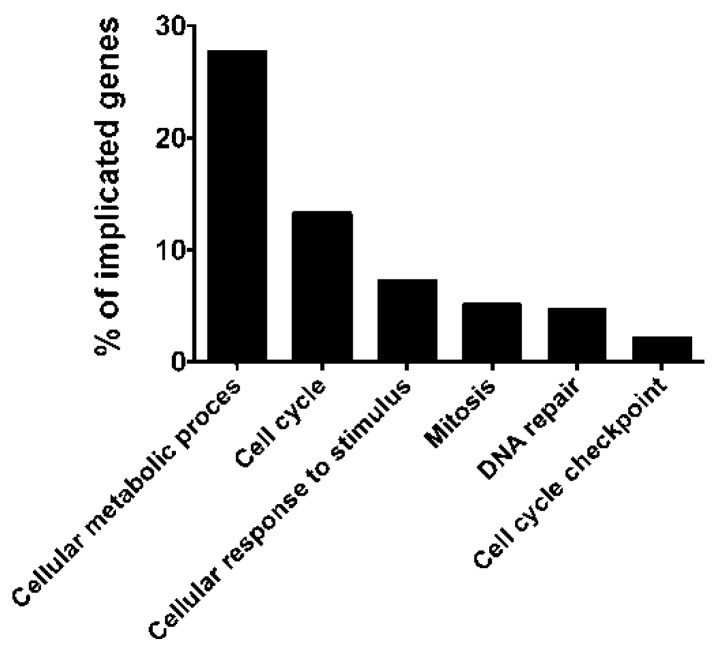
A functional annotation analysis using DAVID Bioinformatic tools v6.7 [24] demonstrated that the target genes for miR-215 were highly represented in the categories of cellular metabolic process (Gene Ontology (GO): 44237), cell cycle (GO: 07049), cellular response to stimulus (GO: 51716), mitosis (GO: 07067), DNA repair (GO: 06281) and cell cycle checkpoint (GO: 00075). The percentages of genes that contributed to representative categories are depicted.

The clinical relevance of miR-215b was not evident, so we sought experimentally-validated target genes of miRNA-215 in miRTarBase or determining the biological significance of miR-215. More than 100 target genes were extracted by miRTarBase and the target genes for which strong evidence existed are summarized in Table 2. Six genes, including WNK lysine deficient protein kinase 1 (*WNK1*), activated leukocyte cell adhesion molecule (*ALCAM*), retinoblastoma 1 (*RB1*), protein tyrosine phosphatase receptor type T (*PTPRT*), activin A receptor type IIB (*ACVR2B*) and catenin beta interacting protein 1 (*CTNNBIP1*), were experimentally validated as genes with strong evidence for being targets (Table 2) [14,15,16,17,18,19,20,21,22,23]. To estimate the biological implication of miR-215 target genes, we then used the DAVID bioinformatic tool for functional annotation of target genes and possible related pathways (*p* < 0.0001). Approximately 30% of the target genes were related to cellular metabolic process, and miR-215 target genes were subsequently determined to be involved in the cell cycle, cellular stress, mitosis, DNA repair and cell cycle checkpoints (Figure 3 and Table S3).

## 3. Discussion

During the last decade, emerging evidence has shown that miRNAs are a key molecular tool for cancer diagnosis and prognosis. A section of tumor tissue or a circulating cell-free fraction, such as plasma, may be used for miRNA analysis for finding novel diagnostic and prognostic markers [12]. In the vast majority of reports dealing with miRNA diagnosis, investigators identified tumor cell-derived miRNA. When a cell-free fraction is used, biologically-relevant miRNA is considered to be exosomal miRNA derived from circulating cancer cells. For miRNA analysis in leukemia, using leukemia cells may indeed be more reliable for determining the biological relevance of these cells than using plasma. However, to find a novel prognostic marker in patients with complete remission, such as STOP-IM patients, we need to use a different type of miRNA analysis. For instance, we recently showed that microRNA-148b, a type of immune miRNA in circulating peripheral blood mononuclear cells (PBMCs), is downregulated in STOP-IM patients [13], in accordance with increased NK cells [6]. This finding indicated the possibility that maintaining UMRD after stopping IM might be due to immune surveillance. In such cases, the miRNA level may reflect the component of circulating immune cells; therefore, miRNA analysis requires careful attention and interpretation in a complete remission state.

To further elucidate the possible role of miRNA in STOP-IM patients, we analyzed plasma miRNA. We and others have reported the clinical relevance of plasma or serum miRNA in hematologic neoplasia [7,8,9,25]. Recent technical advances in exosome separation and characterization enabled analyzing exosomal miRNA rather than plasma or serum miRNA [11]. Theoretically, using exosomal miRNA for analysis would be ideal, but in practical terms, the procedure remains complicated. For this reason, we compared the results obtained from plasma and exosome. Regardless of the template source, plasma miR-215 expression was downregulated in STOP-IM patients (Tables S1 and S2). Our CML patients in the STOP-IM group did not take IM for more than six months before the plasma miRNA assay, indicating that the downregulation of plasma miR-215 in STOP-IM patients was not due to current IM intake. It is well known that approximately 60% of CML patients who discontinue IM relapse within 12 months [3,4,5] and CML stem cells may persist after stopping IM [4]. Since the total IM intake dose was significantly higher in CML patients with low miR-215 levels in the IM group, CML stem cells may be more suppressed, although the prognosis in the CML-IM group did not differ between patients with and without low plasma miR-215 expression. Long-term follow-up of CML-IM patients may clarify this matter.

In the current study, plasma miR-215 level was significantly low in the STOP-IM group, when compared to normal subjects, nevertheless, none of these groups took IM for more than six months. We speculated that the difference of plasma miR-215 level in the IM group (CML patients with UMRD, but maintaining IM therapy) could predict a subset of CML patients who could stop IM safely, but the current study failed to predict the selection of the candidate correctly. Therefore, we searched miR-215 target genes by miRTarBase or those that may be linked to immunological surveillance or residual CML stem cells; miR-215 has previously been demonstrated to be dysregulated in several human malignancies and to be correlated with tumor progression [19,21,26,27]. Meta-analyses have revealed that elevated miR-215 expression is correlated with poor prognosis in renal cell carcinomas [28], glioma [29] and pancreatic cancer [30]. In contrast, decreased expression was associated with advanced disease stage in colorectal cancer [31], and miR-192-5p/215 is a putative tumor suppressor in non-small cell lung cancer [32]. Taken together, miR-215 may potentially be both an oncomiR and a tumor-suppressive miR, with its actual role depending on the type of cancer and the pathway involved. In the current study, it was impossible to conduct a functional analysis of miR-215, such as a reporter assay using specimens from patients in a complete remission state. As an alternative, we utilized bioinformatics tools, such as miRTarBase and DAVID, to identify the miR-215 target gene. miRTarBase includes information on miRNA–target interactions (MTIs), with the collected MTIs having been experimentally validated by reporter assay, Western blot, microarray and next-generation sequencing [33]. Based on miRTarBase, we particularly focused on *RB1* and *ACVR2*, showing strong evidence in this study.

Deng *et al.* [32] reported upregulation of miR-215 in gastric cancer tissues, and functional analysis revealed that inhibition of miR-215 significantly suppressed gastric cancer cell proliferation, possibly via G1 arrest. In addition, miR-215 was able to target *RB1* through its 3′-UTR in gastric cancer cells [32]. Conversely, Braun *et al.* [34] reported that p53 induces upregulation of miR-192, miR-194 and miR-215, and miR-192/miR-215 enhanced CDKN1/p21 levels, colony suppression and cell cycle arrest; therefore, miR-192/miR-215 may suppress carcinogenesis through p21 accumulation and cell cycle arrest. Senanayake *et al.* [19] reported that miR-192, miR-215 and miR-194 had significantly lower expression in nephroblastomas, and they identified the target as activating receptor type 2B (*ACVR2B*), indicating that downregulation of miR-215 may affect an important step in the pathogenesis of renal childhood neoplasms. *ACVR2B* is known to be associated with human embryonic stem cells [35]. Human embryonic stem cells express receptors for nodal (ACVR1B and ACVR2B) and a co-receptor for nodal, and they control *NANOG* expression via *SMAD2*/*SMAD3*; therefore, miR-215 targeting ACVR2B may affect the self-renewal capability of stem cells [36]. Although all of the evidence is provided by databases, DAVID bioinformatics tools clearly showed that miR-215 targets genes in the pathway involved in cell cycle regulation.

The data presented in the current study suggest that the expression level of plasma miRNAs in CML patients who maintained a deep molecular response to IM is lower than in normal subjects. Of the 32 CML patients taking IM at the time of plasma miRNAs examination, 16 patients discontinued IM after the assay, and five have had a molecular relapse. Unfortunately, we observed no significant differences in the plasma miR-215 level or in the clinical data between these two IM groups (data not shown), possibly because of the retrospective study for miRNAs assessment. The plasma miR-215 level in CML patients who had been maintaining molecular remission without imatinib was actually downregulated; therefore, further prospective studies may provide new insight into available biomarkers for therapy-free remission in CML patients.

## 4. Experimental Section

### 4.1. Patients and Samples

Fifty-two consecutive patients with CML, who maintained MR^4.0^ or more by IM therapy, were enrolled in this study. Samples from unselected 20 patients who had sustained UMRD after discontinuing IM (STOP-IM group) were collected more than 6 months after discontinuation [13]. The STOP-IM patients maintained the negativity of nested PCR for more than two years before stopping IM therapy. Thirty-two patients who were receiving IM and maintained less than MR^4.0^ at the time of plasma collection were categorized as the IM group. Twenty-eight healthy volunteers served as the control group. CML patients who had not received treatment and those who did not respond to IM were not included in this study. No significant difference existed in sex or age between the CML groups and healthy volunteers. Molecular response was determined by the *BCR-ABL1* transcript, as reported previously [6,13], and was represented as an International Scale (IS). UMRD was confirmed by the absence of *BCR-ABL1* transcript as determined by nested reverse transcription-polymerase chain reaction (RT-PCR) [13]. This study was approved by the institutional review board of Tokyo Medical University (No. 930, approved 24 June 2008). Written informed consent was obtained from all patients prior to specimen collection, in accordance with the Declaration of Helsinki.

### 4.2. TaqMan Low-Density Array Screening

Total RNA was isolated with the mirVana PARIS kit (Ambion, Austin, TX, USA) from seven randomly-selected healthy volunteers and seven randomly-selected STOP-IM patients. Five hundred microliters of plasma were diluted with 500 µL of binding solution. After a 5-min incubation, 1 µL of 1 nM ath-miR-159 (Hokkaido System Science, Hokkaido, Japan) was added, followed by vortexing for 30 s and incubation on ice for 10 min. Subsequent phenol extraction and cartridge filtration were performed according to the manufacturer’s instructions. The RT reaction and pre-amplification step were set up according to the recommendations. miRNAs were reverse transcribed with Megaplex Prime Pools (Human Pools A v2:1; Thermo Fisher Sciences). The miRNA expression profiles were determined with a TaqMan miRNA Array Human Card A (Thermo Fisher Sciences). To compare the results between plasma miRNA expression and exosomal miRNA expression, we extracted the exosome by Total Exosome Isolation Reagent (Invitrogen, Carlsbad, CA, USA) and then assessed exosomal miRNA expression in a subset of samples, as described previously [11]. Quantitative RT-PCR was performed on an Applied Biosystem 7900 HT thermocycler according to the manufacturer’s recommended program [13]. With the use of SDS2.2 software (Invitrogen) and DataAssist (Thermo Fisher Sciences), the expression of plasma miRNAs was calculated based on cycle threshold (*C*_t_) values normalized to those of ath-miR-159, which was spiked in each plasma sample. Data analysis was done using GeneSpring software (Agilent Technologies). The Benjamin-Hochberg algorithm was used for estimation of false discovery rates, as we reported previously [13].

### 4.3. Real-Time Quantitative RT-PCR for miR-215

We determined the amounts of the individual miRNA by real-time quantitative RT-PCR with a TaqMan MicroRNA Assay (Thermo Fisher Sciences) and the following miRNA-specific stem-loop primer: hsa-miR-215 (hsa-miR-215-4373084). Subsequently, quantitative real-time PCR was performed with an ABI Prism 7000 sequence detection system (Thermo Fisher Sciences). The reaction was initiated by incubation at 95 °C for 2 min, followed by 50 cycles of 95 °C for 15 s and then 60 °C for 1 min. All reactions were run in duplicate. Mean (*C*_t_) values for all miRNAs were quantified with sequence detection system software (SDS Version 1.02, Thermo Fisher Scientific) [13]. The relative expression of miR-215 was calculated by using the ∆*C*t method as reported previously [8]. The spike control (ath-miR-159) was used as an invariant control for plasma miRNA.

### 4.4. Statistical and Bioinformatics Analyses

Data were expressed as means ± standard deviation (SD). Mann-Whitney *U* and χ-square tests were used to determine statistical significance for comparisons between the control and test groups. Multiple groups were compared by one-way analysis of variance (ANOVA). Statistical analysis was done using R software and GraphPad Prism software (Version 5c for Macintosh; GraphPad Software Inc., La Jolla, CA, USA). miRNAs with a ∆*C*_t_ of >1.0 or <−1.0 and *p*-values of >0.05 were deemed as being differentially expressed.

Following identification of differentially-expressed miRNAs, the predicted target genes for these altered miRNAs were subjected to the experimentally-validated miRNA-target interactions database miRTarBase [37]. Moreover, functional annotation of target genes was determined by the Database for Annotation, Visualization and Integrated Discovery (DAVID Bioinformatics Tools v6.7) [24].

## 5. Conclusions

In the current study, we found that plasma miR-215 was downregulated in CML patients who had stopped IM therapy compared to normal subjects. Further studies will reveal the biological and clinical significance of miR-215, including the plasma miR-215 level, and provide further information for treatment-free remission in CML patients. In conclusion, plasma miRNA analysis, which may reflect exosomal miRNA, could be a potent prognostic biomarker in hematologic malignancies that have a current remission status.

## Figures and Tables

**Table 1:** Clinico-hematologic characteristics of chronic myeloid leukemia (CML) patients under imatinib therapy with or without down-regulation of plasma miR-215 expression.

Plasma miR-215 Levels (Number of CML Patients Who Are Taking Imatinib)	<0.6627	>0.6627	*p* Value
	*n* = 22	*n* = 10	
Age (years)	55.91 ± 2.436	58.50 ± 4.634	0.5903
Sex (male/female)	18/4	8/2	1 *
Sokal score (low/intermediate/high)	19/3/0	7/2/1	0.2719 *
Prior interferon therapy (yes/no)	7/15	4/6	0.7026 *
Total IM dose (g)	931.0 ± 65.32	670.5 ± 71.67	0.0229
Daily IM dose (mg)	377.3 ± 11.27	360.0 ± 16.33	0.395
Duration IM (months)	84.05 ± 5.654	65.30 ± 9.656	0.0871
UMRD from diagnosis (months)	53.27 ± 8.855	70.80 ± 22.24	0.3827
Duration of UMRD (months)	41.82 ± 5.283	38.60 ± 9.782	0.7548
International scale percentage at the time of plasma miRNA examination	0.0026 ± 0.0003	0.0031 ± 0.0006	0.4126

IM, imatinib mesylate; UMRD, undetectable minimum residual disease; MR, molecular response; MMR, major molecular response; * determined by the chi-square test.

**Table 2:** Top 10 of the predicted target genes for miR-215 that identified by MiRTarBase.

ID	Target	Validation Methods							Sum	References
		Strong Evidence			Less Strong Evidence					
		Reporter Assay	Western Blot	qPCR	Microarray	NGS	pSILAC	Other		
MIRT005583	*WNK1*	*	*	*				*	4	[14]
MIRT024327	*ALCAM*	*	*	*	*				4	[15,16]
MIRT024341	*RB1*	*	*	*	*				4	[15]
MIRT052961	*ZEB2*		*	*	*				3	[17]
MIRT054778	*PTPRT*	*	*	*					3	[18]
MIRT006340	*ACVR2B*	*	*						2	[19]
MIRT007364	*CTNNBIP1*	*			*				2	[15,20]
MIRT024273	*DTL*				*			*	2	[15,21]
MIRT024412	*GABPB1*				*	*			2	[15,22]
MIRT024495	*SYNGR1*				*	*			2	[15,23]

Asterisks indicate data from validation methods; NGS, next-generation sequencing; pSILAC, stable isotope labeling by amino acid in cell culture.

## Supplementary Materials

Supplementary materials can be found at: http://www.mdpi.com/1422-0067/17/4/570/s1.
